# Atrazine Contamination of Drinking Water and Adverse Birth Outcomes in Community Water Systems with Elevated Atrazine in Ohio, 2006–2008

**DOI:** 10.3390/ijerph15091889

**Published:** 2018-08-31

**Authors:** Kirsten S. Almberg, Mary E. Turyk, Rachael M. Jones, Kristin Rankin, Sally Freels, Leslie T. Stayner

**Affiliations:** 1 Environmental and Occupational Health Sciences Division, School of Public Health, University of Illinois at Chicago, 1603 W. Taylor Street, Chicago, IL 60612, USA; rjones25@uic.edu; 2 Epidemiology and Biostatistics Division, School of Public Health, University of Illinois at Chicago, 1603 W. Taylor Street, Chicago, IL 60607, USA; mturyk1@uic.edu (M.E.T.); krankin@uic.edu (K.R.); sallyf@uic.edu (S.F.); lstayner@uic.edu (L.T.S.)

**Keywords:** atrazine, community water system, low birth weight, preterm birth, small for gestational age, water contamination, endocrine disruptor

## Abstract

Atrazine, a common water contaminant in the U.S., has been associated with adverse birth outcomes in previous studies. This study aimed to determine if atrazine concentrations in drinking water are associated with adverse birth outcomes including small for gestational age (SGA), term low birth weight (term LBW), very low birth weight (VLBW), preterm birth (PTB), and very preterm birth (VPTB). This study included 14,445 live singleton births from Ohio communities served by 22 water systems enrolled in the U.S. Environmental Protection Agency’s Atrazine Monitoring Program between 2006 and 2008. Mean gestational and trimester-specific atrazine concentrations were calculated. Significantly increased odds of term LBW birth was associated with atrazine exposure over the entire gestational period (OR 1.27, 95% CI 1.10, 1.45), as well as the first (OR 1.20, 95% CI 1.08, 1.34) and second trimesters (OR 1.13, 95% CI 1.07, 1.20) of pregnancy. We observed no evidence of an association between atrazine exposure via drinking water and SGA, VLBW, PTB, or VPTB. Our results suggest that atrazine exposure is associated with reduced birth weight among term infants and that exposure to atrazine in drinking water in early and mid-pregnancy may be most critical for its toxic effects on the fetus.

## 1. Introduction

Atrazine is the second most widely used herbicide in the United States, primarily applied to corn and sorghum crops [1]. Much of the concern about atrazine arises from its persistence in soil and its transport to surface and groundwater drinking water sources [2], making it the most commonly detected pesticide in surface water sources in the United States and frequently detected in groundwater sources as well [1,3].

Atrazine is an endocrine disruptor [1,4,5], and while some aspects of the toxic mechanisms are unclear, atrazine disrupts the hypothalamic-pituitary-gonadal axis by inhibiting luteinizing hormone production, increasing aromatase production, and disrupting ovarian function [6,7,8,9]. Low ecologically relevant doses of atrazine have been shown to decrease testosterone levels, reduce spermatogenesis, and alter gonad development in amphibians, leading sometimes to complete chemical feminization of male frogs [10,11]. Exposure to atrazine induces delayed puberty, decreased testosterone and increased estradiol levels, reduced sperm counts, and altered testis architecture [8,12,13,14,15] among male rats and delayed puberty, lengthened estrous cycles, and decreased number of menstrual cycles [9,16] among females.

There is limited epidemiologic evidence of an effect of prenatal exposure to atrazine on adverse birth outcomes in humans. Winchester et al. [17] observed a temporal association between atrazine application and birth defects in an ecologic study in the U.S. Two studies of births in the Midwest have found that increased atrazine levels in drinking water sources is associated with elevated odds of small for gestational age (SGA), with one indicating that the timing of exposure is critical for understanding this association [18,19]. Exposure to atrazine through contaminated drinking water has been associated with increased risk of preterm birth in Kentucky [20] and four Midwestern states [21]. In France, Chevrier et al. [22] reported that the presence of atrazine biomarkers in maternal urine was associated with lower birth weight, length, and head circumference. A recent study found an association between atrazine and both preterm birth and very preterm births in Midwestern counties in which <10% of the population is using private well water [21]. With the exception of the one prospective cohort study in France [22], all previous epidemiological studies of atrazine and birth outcomes have relied on ecologic exposure estimates obtained retrospectively through environmental monitoring data.

The United States Environmental Protection Agency (USEPA) defines the legal limits for water contaminants and water testing schedules, as mandated in the Safe Drinking Water Act. The maximum contaminant level (MCL) for atrazine in drinking water is 3 µg/L [23]. Public water systems are required to test for atrazine quarterly, unless atrazine concentrations are consistently below the MCL, at which point testing can be reduced to once every three years. Those water systems that have atrazine or total combined triazine measurements exceeding 2.6 µg/L in finished water, or 12.5 µg/L in raw water, over a 90-day average are inducted into the Atrazine Monitoring Program (AMP) for 5 years. Community water systems (CWS) in the AMP are required to measure atrazine weekly during the season of peak atrazine use and biweekly throughout the remainder of the year [24].

The primary objective of this study was to examine the association between atrazine concentrations in drinking water and selected adverse birth outcomes among those communities receiving drinking water from community water systems that were part of USEPA’s Atrazine Monitoring Program between 2006 and 2008 in the state of Ohio. This study also aimed to explore the utility of environmental and health data collected through routine monitoring by state and federal agencies for addressing epidemiologic questions, in line with the Centers for Disease Control and Prevention Environmental Public Health Tracking Program [25].

## 2. Materials and Methods

### 2.1. Study Population

This study used birth certificate data from all births occurring within the 22 Ohio communities receiving drinking water from a CWS in the USEPA’s AMP between 2006 and 2008. There were 14,897 births in these cities, of which 14,445 (97%) were singleton births. This analysis was restricted to singleton births as multiple births (e.g., twins and more) have smaller birth weights and shorter gestational periods [26]. The singleton births in this analysis comprised 3.4% of births state-wide (*n* = 428,804) during this time period. Individual-level, de-identified birth certificate data for children born in Ohio were provided by the Ohio Department of Health.

### 2.2. Birth Outcomes

The birth outcomes of interest in this study were small for gestational age (SGA), term low birth weight (term LBW), very low birth weight (VLBW), preterm birth (PTB), and very preterm birth (VPTB). SGA was defined as the smallest 10% of infants, according to birth weight, at each gestational age in the population [26]. Small for gestational age status was calculated using sex- and gestational age-specific national birth weight references [27]. Term LBW was defined as an infant weighing <2500 g at time of delivery among term infants (≥37 weeks gestation). An infant was considered VLBW if it weighed <1500 g at time of delivery, regardless of gestational age. Preterm birth and VPTB were defined as infants delivered prior to 37 and 32 weeks gestation, respectively. Gestational age was based on the reported last normal menstrual period. If the last menstrual period was unknown or implausible, a clinical estimate of gestation was used. All birth outcomes were either reported directly on or were calculated from variables reported on the birth certificates.

### 2.3. Exposure Assessment

Drinking water measurements of atrazine in finished water from 2005 to 2008 were obtained from the USEPA’s AMP public data portal [28] for all 22 AMP water systems in Ohio. Each of these water systems were enrolled in the AMP for all years of the study. We made the assumption that the service boundaries of each CWS in the AMP corresponded to the city limits in which the water system was located. To verify this assumption, we attempted to contact an employee at each AMP water system in Ohio. We successfully reached personnel at 70% of water systems included in this study, and our assumption regarding city and water system boundaries was verified by personnel at 10 of the 15 water systems where contact was made (Ohio Atrazine Monitoring Program Community Water Systems, 2015, personal communications). Personnel at the remaining five water systems were not able to provide this information.

Monthly mean estimates of atrazine in each AMP water system were calculated from the weekly and biweekly samples in the AMP data. Using the mean monthly estimates, we calculated the mean atrazine concentrations for the entire gestational period of the pregnancy (“gestational atrazine”) as well as for each trimester of pregnancy, based on date of birth and gestational age at birth. The limit of detection for atrazine was 0.1 µg/L in 2006 and was 0.05 µg/L in 2007 and 2008 [29]. Measurements below the limit of detection (LOD) were assigned a value of the LOD/2 in this analysis. Surface water was the source for all water systems included in this analysis. Atrazine exposure measures were linked with birth records by the city code of the mother’s residence, which is provided on the birth certificate, as well as the year and month of birth of the infant.

### 2.4. Covariates

The covariates examined in this study included infant sex, maternal age at birth, maternal race/ethnicity, maternal educational attainment, marital status, prenatal care status, socioeconomic status, parity, cigarette use, and maternal pre-pregnancy body mass index (BMI). Maternal age was categorized as <20, 20–34, and ≥35 years of age. Maternal race/ethnicity was defined as non-Hispanic white, non-Hispanic black, Hispanic, and other/unknown. Maternal educational attainment was categorized as less than a high school degree, high school degree, some college, and college degree or higher. Marital status was dichotomized as married or unmarried. The unmarried category includes mothers who responded single, widowed, or divorced. The Kotelchuck index was used to define the adequacy of prenatal care utilization, based on the month of entry into prenatal care and total number of prenatal care visits [30]. Women were categorized as inadequate, intermediate, adequate, or adequate plus—a category that indicates an individual has had more than the recommended amount of prenatal care. Maternal smoking was dichotomized as smoker versus non-smoker. The cigarette use data was non-specific to the window of time including pregnancy. Whether or not the mother was enrolled in the Women, Infant, and Children (WIC) supplemental nutrition program was used as a proxy for low SES. The WIC program is a federally funded nutrition and assistance program for low-income pregnant and post-partum women, infants, and children under the age of five [31]. Maternal pre-pregnancy BMI was categorized according to the Centers for Disease Control and Prevention definitions of underweight, normal, overweight, and obese [32]. Parity was categorized as having had 0, 1, 2, or ≥3 previous live births.

### 2.5. Data Analysis

This is a cross-sectional study of dichotomous birth outcomes among singleton births in Ohio cities served by AMP CWSs. Bivariate associations between atrazine concentrations, outcomes, and covariates were assessed using t-tests for continuous variables, Rao-Scott Chi-Square tests for dichotomous variables, and ANOVA test for covariates with >2 categories. Potential confounders were considered as those variables that were associated with both the exposure measures and outcome measures and were not conceptually in the causal pathway.

We developed generalized estimating equation (GEE) logistic regression models, with an exchangeable working correlation structure and robust standard errors, to estimate the association between atrazine in drinking water and each birth outcome—SGA, term LBW, VLBW, PTB, and VPTB—while accounting for clustering at the city level. Models of continuous and categorical (tertiles) atrazine exposure were tested. Maternal age, maternal race/ethnicity, and year of birth were included in all adjusted models based on a priori knowledge. We assessed confounding throughout the model building process and in an effort to maximize parsimony, we retained only those variables that had a substantial (>10%) effect on the estimate of the effect of atrazine in the models. Final adjusted models were built using the gestational atrazine exposure measure. The covariates identified as confounders in these models were applied to models of trimester-specific atrazine exposure. Third trimester models were not performed for the outcome VLBW because only two of the VLBW births in this population were delivered at full term. Linear GEE regression models of birth weight and gestation in weeks were performed controlling for those covariates identified in the logistic regression model-building procedures. Between 0.5 and 3.5% of observations were not used due to missing data on covariates, exposure, or outcome status. All analyses were performed using SAS^®^, Version 9.4 [33].

We performed a sensitivity analysis to further reduce exposure misclassification by restricting the analysis to only those water systems where we had confirmation from on-site representatives that the service boundaries of the water system corresponded to the city limits in which it was located and that >95% of the population was likely to be receiving their public water from the AMP water system in question (*n* = 10 water systems). Additionally, we restricted the data set to those with a gestational atrazine concentration ≤3 µg/L to evaluate the relationship between atrazine in drinking water and selected birth outcomes when exposure is below permissible levels in public drinking water.

This work was reviewed and approved by the Institutional Review Board of the University of Illinois at Chicago (#2010-0907) and the Institutional Review Board of the Ohio Department of Health (#2016-17).

## 3. Results

### 3.1. Atrazine Concentrations in Drinking Water

**Figure 1:**
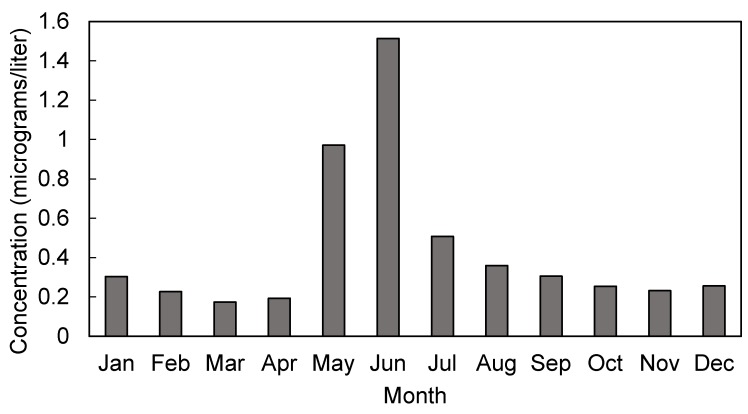
Seasonal variations in mean monthly atrazine concentration (µg/L) in finished water samples from 22 AMP community water systems in Ohio, 2006–2008.

Monthly mean atrazine concentrations in Ohio’s AMP water systems ranged from 0 to 15.7 µg/L between 2006 and 2008 (Table 1). Atrazine measurements followed a sharp seasonal pattern, peaking in the months of May and June (Figure 1). Across all years, monthly mean concentrations were missing in 2.3% of AMP water systems, with annual variation in the missing pattern between 0.5 and 5%. Overall, less than 1% of births were missing an estimate of atrazine exposure during their entire gestation or during any of their trimester estimates of exposure.

### 3.2. Study Population

There were 14,445 live singleton births within the 22 cities which received their public drinking water supply from AMP water systems in Ohio between 2006 and 2008, of which 51% were males (Table 2). The majority of these births were born to mothers who were non-Hispanic white (86%), between 20 and 34 years old (81%), were married (54%), and parous (59%). Half of the births during this time period were born to mothers with a high school degree or less. Overall, 68% of mothers reported adequate plus, intermediate, or adequate prenatal care, but 19% had an unknown level of prenatal care. The proportion of infants born to mothers enrolled in the WIC program in our sample was higher than for the state as a whole (50% versus 42%) as was the proportion of infants born to mothers who reported smoking (35% versus 26%) (not shown). There was a high prevalence of pre-pregnancy obesity (25%) among the mothers in this population. Among live singleton births, 10.3% were SGA, 1.1% were very low birth weight, 9.9% were preterm, and 1.6% very preterm. Among singleton term births, 2.4% were term LBW. Between <1% and 3% of observations were dropped in fully covariate-adjusted models due to missing data on either outcomes, covariates, or exposure estimates.

### 3.3. Regression Analyses

We found weak and statistically non-significant evidence of a positive association between gestational averages of atrazine and SGA in either crude or fully covariate-adjust models (AOR 1.06, 95% CI 0.96, 1.17) (Table 3). In our examination of trimester-specific exposure windows, we similarly observed only a weak association between average atrazine exposure in the first trimesters and SGA (Table 4).

**Figure 2:**
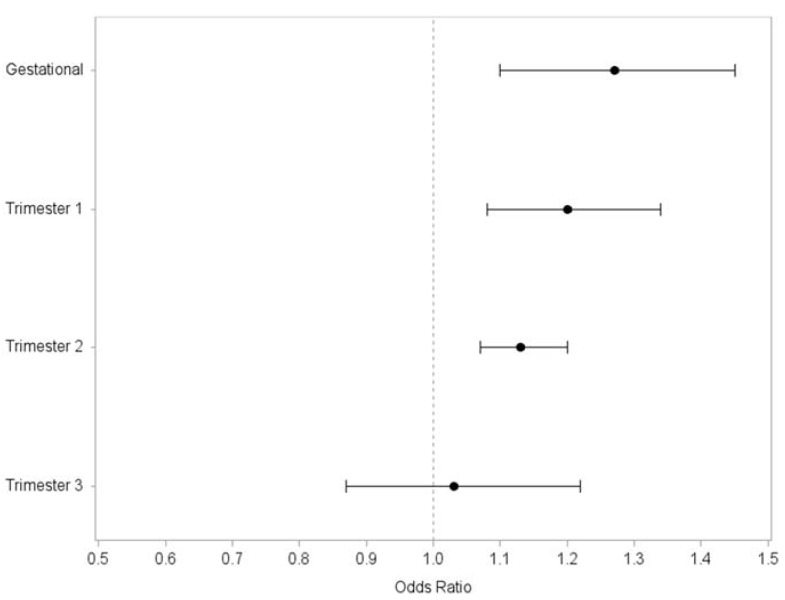
Association between gestational and trimester mean estimates of atrazine exposure in drinking water and term LBW in communities served by water systems enrolled in USEPA’s AMP (*n* = 22) in Ohio, 2006–2008.

Mean gestational atrazine exposure was associated with significantly increased odds of term LBW birth in both crude and adjusted models (Table 3; AOR 1.27, 95% CI 1.10, 1.45). In our models of trimester-specific exposure windows, we observed a significant increase in odds of term LBW birth with increasing atrazine exposure during the first and second trimesters, but not in the third trimester (Table 4; Figure 2). In categorical analyses, we observed a significant increase in odds of term LBW among those in the highest tertile of mean gestational atrazine exposure compared to those in the lowest (Table 5), although odds increased across each tertile of exposure (test for trend *p* = 0.0007).

Results from linear regression analyses indicated that an increasing gestational atrazine exposure was related to decreased birth weight among term infants (−2.7 grams per 1µg/L increase, *p* = 0.77) and gestational age in weeks among all infants (−0.03 weeks per 1 µg/L increase, *p* = 0.62), but these findings were non-significant.

We observed no evidence of an association between gestational or trimester averages of atrazine exposure with odds of VLBW, PTB, or VPTB in this population (Table 3 and Table 4).

### 3.4. Sensitivity Analyses

We restricted the mean gestational and trimester-specific atrazine exposure models to include only those water systems where an on-site representative confirmed that the service boundaries corresponded to the city boundaries. There were 4488 births within these 10 AMP water systems. We observed elevated odds of term LBW per 1 µg/L increase in mean gestational atrazine in this subgroup (AOR 1.16, 95% CI 0.77, 1.74), but the association was not significant (Table 6). We observed no association between gestational atrazine concentrations and SGA or PTB. Covariate-adjusted models did not converge for VPT or VLBW, but no association between atrazine and either outcome was seen in crude models.

Mean exposure to atrazine in the first trimester was significantly associated with an increase in the odds of term LBW in these restricted models (AORT1 1.17, 95% CI 1.03, 1.34) (Table 6). Atrazine exposure during the first trimester was inversely associated with the odds of PTB in crude and adjusted models. No association between atrazine exposure in the second or third trimesters and either SGA, term LBW, or PTB was observed in adjusted models. Third trimester atrazine exposure was not assessed for its relationship to PTB.

In a separate sensitivity analysis of term LBW, we restricted the data set to include only those term births with a mean gestational atrazine concentration ≤3 µg/L (*n* = 12,980), the current MCL set by the USEPA. Used as a continuous measure in the models, mean gestational atrazine exposure was associated with a significant increase in the odds of term LBW (AOR 1.33, 95% CI 1.08, 1.64). Odds of term LBW increased across tertiles of mean atrazine exposure, but only those in the highest tertile of gestational atrazine exposure were at significantly increased odds of term LBW compared to those in the lowest tertile (AOR 1.25, 95% CI 1.10, 1.43).

## 4. Discussion

The aim of this study was to examine the relationship between atrazine exposure during pregnancy and selected adverse birth outcomes among communities that have been served by water systems monitored by the USEPA’s Atrazine Monitoring Program. Furthermore, this research was aimed at elucidating the window of exposure that is most critical for these birth outcomes. In this analysis of all live singleton births within AMP communities in Ohio between 2006 and 2008, we observed a significant increase in odds of term LBW births with increasing atrazine exposure. This association was observed within models of atrazine exposure averaged over the entire gestation of the pregnancy. Furthermore, our results suggest that atrazine exposure within the first and second trimesters of pregnancy, but not during the third trimester, are associated with term LBW, indicating that exposure to atrazine in drinking water in early and mid-pregnancy may be most critical for its toxic effects on the fetus. We observed no significant evidence of an association between atrazine exposure via drinking water and SGA, VLBW, PTB, or VPTB.

The exact mechanism through which atrazine would reduce birth weight is not well understood. Findings from rat models showing reduced pup weight after in utero exposure to atrazine lend biologic plausibility to our findings [34,35]. Our findings are consistent with previous epidemiologic research which has shown an inverse relationship between atrazine exposure and birth weight [22], but conflicts with another study which found no association between atrazine exposure via drinking water and low birth weight in a population of infants in Brittany, France [36]. While previous studies have shown evidence of an association between atrazine exposure and small for gestational age and preterm birth [21], we found no evidence of these associations in our study of singleton births occurring within communities served by AMP water systems in Ohio from 2006 to 2008.

Reduced birth weight has serious public health impacts. The risk of neonatal mortality is highest among the smallest and largest infants, as measured by birth weight. This same pattern of increased risk is seen later in life as well, with a reversed “J” shape association between birthweight and cardiovascular disease and all-cause mortality [26]. Our findings suggest that the morbidity and mortality burden from this adverse birth outcome can be lessened through reducing gestational exposures to atrazine in drinking water.

Water systems are enrolled in the AMP as a result of repeated exceedances of the 3 µg/L MCL for atrazine, but only 4% of samples from the water systems in this study exceeded the MCL. Our findings are unchanged when we remove those observations for which gestational atrazine estimates exceeded the MCL. While further epidemiologic research is needed, these results suggest that the current MCL for atrazine may not be protective against some adverse birth outcomes such as term low birth weight.

Most previous epidemiologic studies of atrazine and birth outcomes have been limited by ecologic exposure and outcome assessment. In the present study, birth outcomes and covariates were assessed at the individual level from birth certificates, providing more accurate outcome ascertainment and robust control of confounding. Atrazine exposure was estimated at the water system level in this study, which offers substantial refinement of exposure classification from the ecologic measurements that combine observations across multiple CWSs used in some of the prior studies [18,20,36]. Furthermore, the sampling frame under the USEPA’s Atrazine Monitoring Program is more intensive than the frame for low-risk CWSs, which allows more robust determination of monthly atrazine concentrations and minimizes the number of months missing data in this analysis. Despite the reduction in exposure misclassification by estimating atrazine for each unique water system, we remain unable to account for personal drinking water behaviors (e.g., use of bottled water or filters), which can substantially influence an individual’s exposure. We lacked data on atrazine exposure from other sources such as diet, although atrazine residue is not often detected on food products and is not considered to be a significant contributor to overall atrazine exposure in the general population [37]. Furthermore, this study assessed the relationship between exposure to one contaminant and multiple birth outcomes, which does not address the fact that drinking water contains varying levels of multiple contaminants.

We made an assumption that the service boundaries of the AMP water systems in this study corresponded to the geographic boundaries of the city in which each was located. For nearly half of these water systems, we received verbal confirmation from treatment plant operators and water system managers that this was in fact the case. We performed a sensitivity analysis by restricting the gestational atrazine models to only these confirmed water systems to attempt to further reduce exposure misclassification. In this sub-group analysis, we saw consistent magnitude and direction of association between atrazine and term low birth weight compared with the full sample, but lacked sufficient numbers to detect a significant increase in odds of this rare outcome.

Our outcome and covariate data originated from birth certificates. The reliability of birth certificate data, however, varies widely by data element. Overall, the Ohio birth certificate data contained low levels (<3%) of missing data on the key covariates used in these analyses. A notable exception is the high level of missing data on prenatal care (26%). Those who were missing data on their prenatal care status were more likely to be non-Hispanic black, “Other” race/ethnicity, and young. We chose to only use those covariates that are considered to be well-reported and highly accurate on birth certificates, such as maternal age, race/ethnicity, marital status, parity, plurality, infant gender, birth weight, and gestational age [38,39,40,41,42,43,44,45].

We lacked information on whether or not the mothers of the infants in these analyses had moved at any point during their pregnancy and assumed that the residence listed on the birth certificate was the residence throughout the entire pregnancy. Rates of pregnancy mobility are estimated between 12 and 32% [46,47,48,49], and vary by geography and demographic factors.

Our study was restricted to a small percentage of births (3%) in the state of Ohio for this analysis of AMP water systems. The population in these AMP communities differed from the state population in important ways. A much higher percentage of infants were born to mothers who were non-Hispanic white (86%) compared to the state as a whole (76%). Additionally, these AMP communities had a higher proportion of births from women enrolled in WIC (50% versus 42%) and who reported smoking (35% versus 26%). The small sample size relative to the state population and the demographic differences between the AMP communities and the state as a whole limit the generalizability of the study results. Ideally, future research on the association between atrazine in drinking water and adverse birth outcomes would include a representative sample of births to increase the generalizability of study findings.

Despite these limitations, the study had several notable strengths. The exposure estimates used in this study are highly geographically and temporally refined, which allowed specific exposure windows, such as trimesters, to be examined. The large number of births included in this study allowed the examination of two rare outcomes, very preterm birth and very low birth weight, which have not been reported previously. Furthermore, this study also benefited from individual-level data on important covariates.

Our findings suggest that additional epidemiologic research should examine the reproductive effects of exposure to atrazine in areas of relatively low contaminant exposure. Ideally, future research would employ biomarkers of exposure or individual assessment of drinking water exposures rather than relying on the ecologic exposure measures presented in these analyses. Despite the limitations in the exposure ascertainment, our findings demonstrate that linking environmental monitoring data with health outcomes data, such as vital statistics databases, holds promise for identifying potential associations, which can subsequently be investigated using with more refined exposure and outcome ascertainment.

## 5. Conclusions

We found an association between atrazine concentrations in drinking water and the odds of term LBW births within communities served by water systems enrolled in USEPA’s Atrazine Monitoring Program in Ohio. This is the first study to show such an association for term LBW by linking maternal residence to a specific water system. Water systems are enrolled in the AMP as a result of repeated exceedances of the 3 µg/L MCL for atrazine, but only 4% of samples from the water systems in this study exceeded the MCL. We observed an increase in the odds of term LBW births among those with average gestational atrazine below the current MCL. While further epidemiologic research is needed, these results suggest that the current MCL for atrazine may not be protective against some adverse birth outcomes such as term LBW.

## Figures and Tables

**Table 1:** Summary of monthly mean atrazine concentrations reported in finished drinking water by Atrazine Monitoring Program (AMP) community water systems (*n* = 22) in Ohio, 2006–2008.

Year	Geometric Mean, GSD (µg/L)	Median Concentration (µg/L)	Minimum Concentration (µg/L)	Maximum Concentration (µg/L)	Percent Missing ^a^ (%)
2006	0.29, 3.04	0.17	0.00	7.22	1.5
2007	0.15, 3.43	0.16	0.03	4.23	0.4
2008	0.16, 4.35	0.17	0.03	15.66	4.9

^a^ Percentage of community water systems missing monthly mean atrazine concentration. GSD: geometric standard deviation.

**Table 2:** Distribution of demographic and economic covariates across the population of live singleton births (*n* = 14,445) and the prevalence of outcomes by covariates in Atrazine Monitoring Program communities in Ohio, 2006–2008.

Variable	*n* (%)	SGA	Term LBW	VLBW	PTB	VPTB
		%	%	%	%	%
**Gender**						
Male	7431 (51)	9.9	1.9	1.1	10.5	1.7
Female	7014 (49)	10.6	3.4	1.1	9.3	1.6
**Race/Ethnicity**						
Non-Hispanic white	12,471 (86)	9.7	2.4	1.0	9.5	1.5
Non-Hispanic black	1068 (7)	17.8	6.2	2.4	13.9	2.9
Hispanic	689 (5)	8.9	1.8	1.5	10.3	2.0
Other	217 (2)	9.7	3.2	1.4	13.4	2.3
**Maternal Age at Birth**						
<20	1811 (13)	15.4	4.0	1.7	13.2	2.7
20−34	11,710 (81)	9.7	2.5	1.0	9.4	1.4
35+	923 (6)	7.8	2.7	1.5	10.1	2.1
**Maternal Education**						
High School or less	7203 (50)	12.8	3.1	1.4	11.4	2.0
Some College/Degree	7203 (50)	7.7	2.2	0.8	8.4	1.3
**Maternal Smoking**						
Yes	4995 (35)	7.9	3.9	1.4	11.5	2.1
No	9449 (65)	14.7	2.0	0.9	9.1	1.4
**Prenatal Care**						
Inadequate	1861 (13)	13.2	3.8	1.0	11.2	1.9
Intermediate/Adequate	5970 (41)	9.7	1.6	0.3	3.0	0.4
Adequate Plus	3902 (27)	9.6	3.8	1.4	17.5	2.2
Unknown	2712 (19)	10.6	3.0	2.5	13.3	3.3
**WIC use** ^a^						
Yes	7064 (50)	12.6	3.3	1.2	9.1	1.8
No	7108 (50)	7.9	2.0	1.0	10.6	1.4
**Pre-pregnancy BMI**						
Underweight	673 (8)	17.8	6.8	1.2	11.6	1.5
Normal	6664 (47)	11.2	2.8	1.1	9.9	1.7
Overweight	3170 (22)	8.8	2.0	1.0	9.2	1.4
Obese	3781 (26)	8.6	2.4	1.2	10.2	1.6
**Parity**						
0	5892 (41)	12.0	3.2	1.3	10.0	1.8
1	4515 (32)	8.3	2.3	0.9	9.0	1.4
2	2334 (16)	9.3	2.0	1.0	9.6	1.5
≥3	1483 (10)	11.2	2.9	0.7	12.8	1.7
**Marital Status**						
Married	7765 (54)	7.1	1.8	0.8	8.5	1.0
Unmarried	6680 (46)	13.9	3.8	1.5	11.6	2.3

^a^ WIC use indicates participation in the Women, Infant and Children nutrition and assistance program. SGA: small for gestational age; Term LBW: term low birth weight; VLBW: very low birth weight; PTB: preterm birth; VPTB: very preterm birth.

**Table 3:** Associations ^a^ between gestational mean atrazine concentrations in drinking water and SGA, term LBW, VLBW, PTB, and VPTB in AMP communities (*n* = 22) in Ohio, 2006–2008.

Outcome	Model	*n*	OR ^g^ (95% CI)
SGA ^b^	Crude	13,942	0.99 (0.88, 1.12)
	Adjusted	13,942	1.06 (0.96, 1.17)
Term LBW ^c^	Crude	12,567	1.15 (1.01, 1.31)
	Adjusted	12,567	1.27 (1.10, 1.45)
VLWB ^d^	Crude	14,089	0.90 (0.50, 1.60)
	Adjusted	14,089	0.81 (0.47, 1.39)
PTB ^e^	Crude	14,098	1.01 (0.89, 1.14)
	Adjusted	14,098	0.99 (0.88, 1.11)
VPTB ^f^	Crude	14,349	1.15 (0.86, 1.55)
	Adjusted	14,349	1.11 (0.81, 1.51)

^a^ All adjusted models included maternal race/ethnicity, maternal age, and birth year a priori. ^b^ Small for gestational age defined as the smallest 10% of infants, according to birth weight, at each gestational age in the population. In addition to a priori variables, the final model for SGA included maternal education, WIC status, marital status, maternal pre-pregnancy BMI. ^c^ Term low birth weight is defined as < 2500 g among term births (≥ 37 weeks gestation). In addition to a priori variables, the final model for term LBW included infant sex, maternal education, WIC status, marital status, maternal smoking status, and maternal pre-pregnancy BMI. ^d^ Very low birth weight is defined as < 1500 g at time of delivery. In addition to a priori variables, the final model for term LBW included maternal education, marital status, and parity. ^e^ Preterm births defined as infants delivered before 37 weeks gestation. In addition to a priori variables, the final model for PTB included maternal education, maternal smoking status, and parity. ^f^ Very preterm births defined as infants delivered before 32 weeks gestation. In addition to a priori variables, the final model for PTB included maternal marital status. ^g^ Odds ratios reflect increase in odds per 1 µg/L increase in atrazine in drinking water.

**Table 4:** Associations ^a^ between trimester-specific atrazine concentrations (µg/L) and SGA, term LBW, VLBW, PTB and VPTB among AMP communities (*n* = 22) in Ohio, 2006–2008.

Outcome	Model	*n*	OR ^g^ (95%CI)
**First Trimester**			
SGA ^b^	Crude	14,022	1.02 (0.95, 1.09)
	Adjusted	14,022	1.04 (0.98, 1.11)
Term LBW ^c^	Crude	12,647	1.14 (1.01, 1.28)
	Adjusted	12,647	1.20 (1.08, 1.34)
VLWB ^d^	Crude	14,170	1.09 (0.86, 1.37)
	Adjusted	14,170	1.07 (0.86, 1.34)
PTB ^e^	Crude	14,179	1.01 (0.90, 1.13)
	Adjusted	14,179	0.99 (0.90, 1.10)
VPTB ^f^	Crude	14,432	1.11 (0.81, 1.53)
	Adjusted	14,432	1.11 (0.81, 1.53)
**Second Trimester**			
SGA ^a^	Crude	14,002	0.97 (0.93, 1.00)
	Adjusted	14,002	0.99 (0.96, 1.02)
Term LBW	Crude	12,647	1.06 (0.98, 1.14)
	Adjusted	12,647	1.13 (1.07, 1.20)
VLWB	Crude	14,148	0.79 (0.55, 1.14)
	Adjusted	14,148	0.76 (0.51, 1.13)
PTB	Crude	14,156	0.99 (0.95, 1.04)
	Adjusted	14,156	0.99 (0.95, 1.04)
**Third Trimester**			
SGA ^a^	Crude	12,648	0.98 (0.87, 1.10)
	Adjusted	12,648	1.00 (0.93, 1.08)
Term LBW	Crude	12,647	0.97 (0.80, 1.16)
	Adjusted	12,647	1.03 (0.87, 1.22)

^a^ All adjusted models included maternal race/ethnicity, maternal age, and birth year a priori. ^b^ Small for gestational age defined as the smallest 10% of infants, according to birth weight, at each gestational age in the population. In addition to a priori variables, the final model for SGA included maternal education, WIC status, marital status, and maternal pre-pregnancy BMI. ^c^ Term low birth weight is defined as <2500 g among term births (≥37 weeks gestation). In addition to a priori variables, the final model for term LBW included infant sex, maternal education, WIC status, marital status, maternal smoking status, and maternal pre-pregnancy BMI. ^d^ Very low birth weight is defined as <1500 g at time of delivery. In addition to a priori variables, the final model for term LBW included maternal education, marital status, and parity. ^e^ Preterm births defined as infants delivered before 37 weeks’ gestation. In addition to a priori variables, the final model for PTB included maternal education, maternal smoking status, and parity. ^f^ Very preterm births defined as infants delivered before 32 weeks’ gestation. In addition to a priori variables, the final model for PTB included maternal marital status. ^g^ Odds ratios reflect increase in odds per 1 µg/L increase in atrazine in drinking water.

**Table 5:** Association ^a^ between term low birth weight births and tertiles of gestational atrazine exposure among live singleton births in AMP communities (*n* = 22) in Ohio, 2006–2008.

Tertile	Exposure Range (µg/L)	OR ^a^ (95% CI)
1	0–0.1537	Ref.
2	0.1538–0.4622	1.11 (0.92, 1.34)
3	0.4623–5.9337	1.26 (1.11, 1.44)
		*p* for trend = 0.0007

^a^ Model adjusted for maternal race/ethnicity, maternal age, birth year, infant sex, maternal education, WIC status, marital status, maternal smoking status, and maternal pre-pregnancy BMI.

**Table 6:** Associations ^a^ between gestational and trimester-specific mean concentrations of atrazine in drinking water and term LBW among AMP communities with verified service boundaries (*n* = 10) in Ohio, 2006–2008.

Exposure	Model	*n*	OR ^b^ (95% CI)
Gestational mean	Crude	3929	1.04 (0.62, 1.72)
	Adjusted	3929	1.16 (0.77, 1.74)
First trimester	Crude	3961	1.13 (0.96, 1.33)
	Adjusted	3961	1.17 (1.03, 1.34)
Second trimester	Crude	3961	0.95 (0.77, 1.17)
	Adjusted	3961	1.01 (0.83, 1.22)
Third trimester	Crude	3961	0.96 (0.66, 1.40)
	Adjusted	3961	1.01 (0.72, 1.41)

^a^ Term low birth weight is defined as <2500 g among term births (≥37 weeks’ gestation). All adjusted models included maternal race/ethnicity, maternal age, birth year, infant sex, maternal education, WIC status, marital status, maternal smoking status, and maternal pre-pregnancy BMI. ^b^ Odds ratios reflect increase in odds per 1 µg/L increase in atrazine in drinking water.
